# Physiological responses of the abalone *Haliotis discus hannai* to daily and seasonal temperature variations

**DOI:** 10.1038/s41598-019-44526-3

**Published:** 2019-05-29

**Authors:** Hee Yoon Kang, Young-Jae Lee, Woo-Young Song, Tae-Ik Kim, Won-Chan Lee, Tae Young Kim, Chang-Keun Kang

**Affiliations:** 1 Gwangju Institute of Science and Technology, School of Earth Sciences and Environmental Engineering, Gwangju, 61005 Republic of Korea; 2 National Institute of Fisheries Science, Southwest Sea Fisheries Research Institute, Gyeongnam, 53085 Republic of Korea; 3 National Institute of Fisheries Science, Marine Environment Research Division, Busan, 46083 Republic of Korea

**Keywords:** Proteomics, Ecophysiology

## Abstract

Organisms inhabiting tidal mixing-front zones in shallow temperate seas are subjected to large semidiurnal temperature fluctuations in summer. The ability to optimize energy acquisition to this episodic thermal oscillation may determine the survival, growth and development of these ectotherms. We compared the physiological and molecular responses of *Haliotis discus hannai* cultivated in suspended cages to fluctuating or stable temperature conditions. Several physiological indicators (respiration, excretion rates and O:N) were measured in both conditions, and alterations in the proteome during thermal fluctuations were assessed. No summer mortality was observed in abalone cultivated in fluctuating temperatures compared with that at stable high temperatures. Metabolic rates increased sharply during stable warm summer conditions and fluctuated in accordance with short-term temperature fluctuations (20–26 °C). Ammonia excretion rates during acute responses were comparable in both conditions. When abalone were exposed to fluctuating temperatures, enzyme activities were downregulated and structure-related protein expression was upregulated compared with that at an acclimation temperature (26 °C), highlighting that exposure to low temperatures during fluctuations alters molecular processes. Our results reveal that modulation of physiological traits and protein expression during semidiurnal thermal fluctuations may buffer abalone from the lethal consequences of extreme temperatures in summer.

## Introduction

The temperature dependence of physiological processes in marine ectotherms is well recognized^1,2^. The metabolic rate of an animal is a primary physiological process that determines its energy requirements. A prolonged condition of energy imbalance, in which the metabolic energy expenditure of an animal exceeds its energy acquisition (or production), can inhibit growth, reproduction and performance, and even lead to death^3,4^. Because ectothermic organisms have the physiological plasticity to adapt to thermal variation in their habitats^5,6^, much attention has been paid to the mechanisms of the physiological adjustments made during their acclimation (or acclimatization) to changes in environmental temperatures. In this context, while some marine mollusks maintain metabolic rates at relatively constant levels during thermal acclimation^7,8^, other ectotherms show a sharp increase in metabolic costs even after thermal acclimation^2,9^. In both cases, an acute increase in environmental temperatures results in increases in the metabolic rates of the organisms. Despite a general consensus among scientists about the thermal dependence of metabolic rate in marine ectotherms, the acclimatory adjustment of their physiological activities in response to a short-term periodic (e.g. diel or semidiurnal) fluctuation in water temperature is still debated^10^.

Marine ectotherms in various temperate coastal sea areas, i.e. tidal flats, tide pools and tidal-mixing fronts, can experience large short-term thermal fluctuations. The responses to short-term cyclic thermal fluctuations vary greatly between ectotherm species and/or physiological processes^10–12^. Such thermally fluctuating conditions can increase the metabolic energy demands of some ectotherms, and even result in a failure to maintain physiological processes and performance traits^10,13,14^. However, many ectotherms can adjust their physiological rates to reduce the thermal sensitivity of their metabolism and mitigate energetic demands in response to short-term fluctuations in temperature^10,15–17^, thus increasing their thermal tolerance by reducing the metabolic cost of exposure to high temperature^10,18,19^. The resultant physiological compensation for either the increased metabolic demands or the reduced metabolic costs occurring during wide thermal fluctuations can accelerate the organism’s growth rate compared with those in corresponding thermally stable conditions^20,21^. Furthermore, the degree of temperature fluctuation that an organism experiences in its habitats may also be an important determinant of its physiological adjustment to short-term thermal variations^21,22^. Therefore, although ectotherms that inhabit habitats exposed to short-term thermal variations require physiological plasticity to buffer the energetic demands from the thermally variable conditions to ensure their survival, growth and development^10,22^, it remains difficult to generalize the patterns of their physiological responses to thermal variations because thermal tolerance windows differ among species^4^.

Here, we examined the physiological and molecular responses of the Pacific abalone, *Haliotis discus hannai*, to thermal variations and determined whether it could adjust its physiological processes to reduce energetic costs under variable (semidiurnal) compared with stable (seasonal) daily temperature conditions. Abalone aquaculture in the shallow coastal seas around Wando Island off the southwestern coast of the Korean Peninsula has been expanding since 1970, and the annual production reached 12,000 tonnes in 2016 (https://www.mof.go.kr/statPortal/). The southwestern area of the sea south of Korea is characterized by a strong tidal front between the bottom cold water of the Yellow Sea and the stable offshore Tsushima Warm Current water^23,24^, and hence is greatly influenced by the cyclic intrusion of cold water toward the coastline during the flood tide after vertical mixing of the surface warm water with the bottom cold water around the tidal front^25^. Therefore, abalone cultivated in suspended cage nets are exposed to large semidiurnal fluctuations in environmental temperature, especially in summer. In recent years, there have been frequent incidences of mass mortality of the abalone cultivated in this area during summer, leading to speculation about possible effects of global warming or an acute temperature variation^25–28^. Because of the high commercial value of the abalone and the feedback effects of farming in ambient natural environments^29^, the capacity of the abalone to adjust their physiological processes to short-term as well as long-term variations in environmental temperatures has become the center of attention.

**Figure 1:**
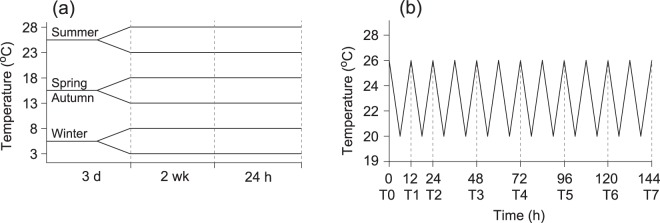
Experimental design and water temperature regimes. (**a**) Stable daily thermal fluctuation in different seasons. For stable daily temperature (seasonal variation) treatments, water temperatures were adjusted to 3 °C and 8 °C for winter conditions, 13 °C and 18 °C for spring/autumn conditions, and 23 °C and 28 °C for summer conditions, respectively. Because of the seasonal variation in water temperature, physiological measurements were conducted 2 weeks after acclimation to 3, 8, 13, 18, 23 and 28 °C, respectively, depending on the season. (**b**) Semidiurnal temperature-fluctuation treatment and timing of abalone tissue sampling. Specimens for the daily fluctuating treatment were kept under a summer temperature (26 °C) and then the experimental temperatures were set to range between 20 °C and 26 °C, based on previous observations of daily temperature variability in the abalone culture cages. The water temperature in the water baths fluctuated periodically at an interval of 6 h and physiological measurements for the fluctuating-temperature treatments were performed for 144 h. Specimens for proteomic analysis were randomly sampled at time points of 0, 12, 24, 48, 72, 96, 120 and 144 h (T0–T7, respectively) after the experiment started.

The measurement of the biological responses of ectotherms to fluctuating thermal conditions is therefore crucial to evaluate how they maintain fitness under these conditions. Abalone cultivated in this shallow coastal water should be ideal for investigating the physiological responses to semidiurnal and seasonal thermal variations. Given the expected acute responses of abalone to the prevalence of fluctuating thermal conditions and their mortality across a widespread cultivation area around Wando Island in summer, we hypothesized that the physiological responses of abalone to thermal fluctuations would limit their ability to maintain growth and performance compared with those acclimated to stable thermal conditions. To test this hypothesis, we evaluated the thermal dependence of the metabolic rate of the abalone in response to daily temperature fluctuations under controlled conditions, comparing this with the responses of those acclimated to seasonal temperature conditions (Fig. 1). We also measured the catabolic breakdown of proteins stored in tissues as a metabolic fuel source for energetic costs. We then evaluated changes in the proteome of *H. discus hannai* that are indicative of cellular stresses to determine whether the organism-level responses to short-term thermal variations correspond with the molecular responses^4^. We anticipated that proteome traits associated with metabolic responses to semidiurnal temperature fluctuation would allow us to assess the abalone’s capacity to adapt to short-term fluctuation and thereby maintain their fitness^30,31^. Specifically, we combined physiological analysis with isobaric tags for relative and absolute quantitation (iTRAQ)-based quantitative mass spectrometry (MS) of the abalone proteome to highlight the regulatory molecular mechanisms behind their physiological performance in response to semidiurnal temperature fluctuation^32,33^.

## Results

### Water temperature

**Figure 2:**
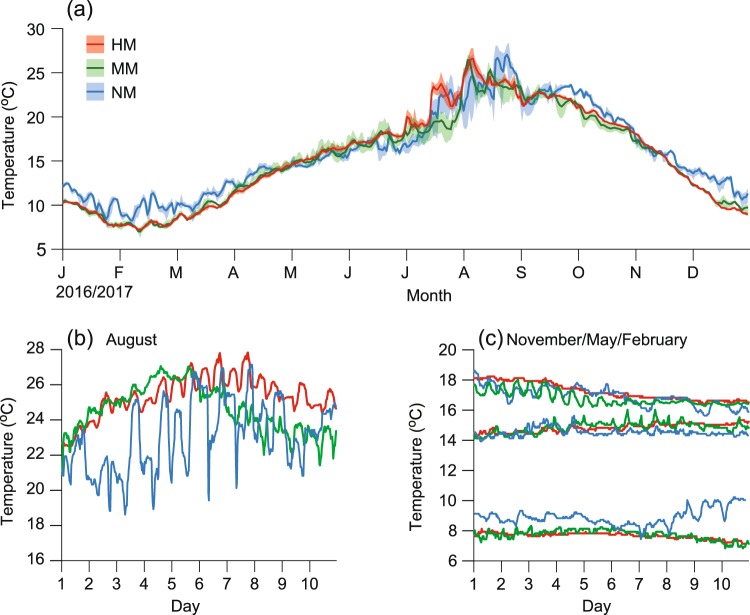
Thermal variability in the abalone-rearing cages. Temperature profile from a data logger in 2017 deployed in the cages of three abalone-culture locations around the archipelago off southwestern Korea. (**a**) Seasonal variabilities of water temperatures in cages representing three localities of high, moderate and no mortality (HM, MM and NM; green, red and blue, respectively). Thick lines represent daily mean temperatures; colored areas represent the range of daily maximum and minimum temperatures. (**b**) Daily fluctuations of water temperatures in the three cages in August, the annually warmest period. (**c**) Small daily temperature fluctuations in the three cages in May, November and February, respectively.

Three abalone-culture cages recorded about 13%, 2% and no mortality, respectively, in each month during the summer (August–October 2017), which allow us to define the high-mortality (HM), the moderate-mortality (MM) and the control no-mortality (NM) cages. The daily mean seawater temperature at the sampling sites showed a seasonal pattern typical of the temperate zone, with a maximum in summer (August) and a minimum in winter (February, Fig. 2a). The HM, MM and NM cages displayed daily mean temperature ranges of 7.2 to 26.6 °C, 7.0 to 26.4 °C and 8.3 to 27.1 °C, respectively. The three sites recorded average daily fluctuations of 1.7 ± 0.4 °C (range: 1.2 to 2.2 °C), 1.8 ± 0.8 °C (0.9 to 3.7 °C) and 5.0 ± 1.9 °C (2.1 to 7.5 °C), respectively, in the warmest period of August (Fig. 2b). The daily temperature fluctuation was largest at the NM of all cages (ANOVA, *F*_2,27_ = 23.61, *P* < 0.001; Tukey test, *P* < 0.05). Except for the summer months, the daily water temperatures were fairly stable with ranges of <1 °C in all the cages (Fig. 2c).

### Metabolic rate of *H. discus hannai*

**Figure 3:**
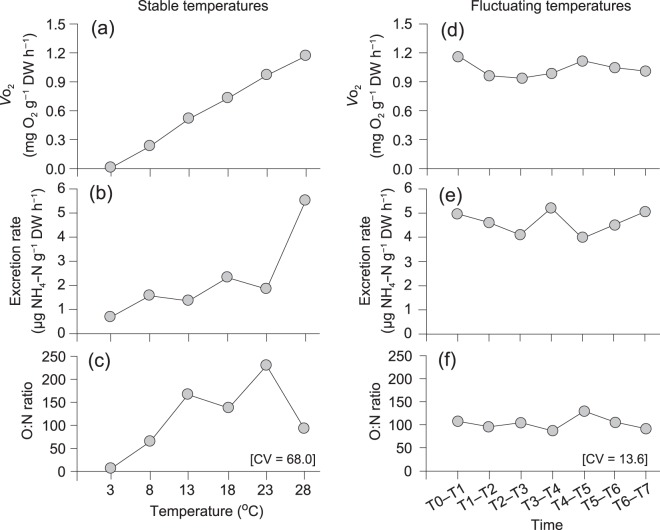
Rates of oxygen consumption (**a**,**d**) and ammonia excretion (**b**,**e**), and O:N ratios (**c,f**) of *Haliotis discus hannai* measured under stable and fluctuating temperature conditions, respectively. Stable temperatures represent stable daily thermal fluctuation in different seasons (see Fig. 1). Physiological measurements were conducted 2 weeks after acclimation to 3, 8, 13, 18, 23 and 28 °C, respectively. Fluctuating temperatures represent semidiurnal temperature-fluctuation (range: 20–26 °C) treatment. Physiological measurements for the fluctuating temperature treatments were performed over 144 h. T0–T7 indicates each time point of 0, 12, 24, 48, 72, 96, 120 and 144 h (T0–T7, respectively) after the experiment started at the acclimated temperature (26 °C). Data points are recalculated regression intercepts from Table 1. CV, coefficient of variation.

Highly significant and positive relationships were found between the rate of oxygen consumption (*V*o_2_, mg O_2_ h^−1^) and dry tissue weight (DW, g) under both stable and fluctuating temperature conditions (Table 1). For the stable daily temperature (seasonal variation) treatments, an analysis of covariance (ANCOVA) showed no significant difference among the slopes of any of the *V*o_2_–DW regressions at the six experimental temperatures (*F*_5,42_ = 1.140, *P* = 0.354), which yielded a common slope of 0.660 (±0.068). However, there was a significant difference among the intercepts of the six regressions (*F*_5,47_ = 446.9, *P* < 0.001). A Bonferroni *post hoc* test demonstrated a significant effect of temperature on oxygen consumption (*P* < 0.05), with a linear increase in *V*o_2_ with increasing test temperature (Fig. 3a).

**Table 1:** Regression coefficients between physiological rate (*V*o_2_, mg h^−1^ for oxygen consumption; ${V}_{N{H}_{4}\mbox{--}N}$, μg h^−1^ for ammonia excretion) and dry tissue weight (DW, g) for *Haliotis discus hannai* according to the allometric equation $V{{\rm{o}}}_{2}\,{\rm{or}}\,{V}_{N{H}_{4}\mbox{--}N}=aD{W}^{b}$ under stable and fluctuating daily temperature treatments.

	Treatments	Temperature (°C)	Slope *b*	Intercept *a*	*r*	$\bar{{\boldsymbol{b}}}$	$\bar{{\boldsymbol{a}}}$
*V*o_2_	*Stable*						
	Winter	3	0.937	0.006	0.823	0.660 ± 0.068	0.010^*a*^
		8	0.635	0.243	0.737		0.232^*b*^
	Spring/fall	13	0.640	0.534	0.887		0.517^*c*^
		18	0.834	0.541	0.975		0.730^*d*^
	Summer	23	0.467	1.194	0.771		0.969^*de*^
		28	0.488	1.372	0.862		1.167^*e*^
	*Fluctuating*						
	T0–T1	20–26	0.718	0.947	0.934	0.589 ± 0.038	1.168
	T1–T2	20–26	0.646	0.878	0.940		0.962
	T2–T3	20–26	0.528	1.107	0.907		0.936
	T3–T4	20–26	0.469	1.198	0.890		0.987
	T4–T5	20–26	0.396	1.529	0.925		1.118
	T5–T6	20–26	0.525	1.158	0.892		1.045
	T6–T7	20–26	0.652	0.912	0.931		1.010
${V}_{N{H}_{4}\mbox{--}N}$	*Stable*						
	Winter	3	0.901	0.394	0.734	0.616 ± 0.081	0.678^*a*^
		8	0.476	2.200	0.885		1.568^*bc*^
	Spring/fall	13	0.466	1.850	0.768		1.355^*b*^
		18	0.539	2.837	0.743		2.322^*c*^
	Summer	23	0.845	1.540	0.788		1.844^*bc*^
		28	0.575	6.125	0.743		5.509^*d*^
	*Fluctuating*						
	T0–T1	20–26	0.510	4.661	0.787	0.471 ± 0.052	4.966
	T1–T2	20–26	0.480	4.538	0.731		4.606
	T2–T3	20–26	0.569	3.497	0.748		4.098
	T3–T4	20–26	0.377	6.062	0.845		5.207
	T4–T5	20–26	0.561	3.416	0.874		3.954
	T5–T6	20–26	0.488	4.383	0.792		4.506
	T6–T7	20–26	0.315	6.509	0.761		5.055

Nine individuals were used for each treatment. All regressions were significant at *P* < 0.001. $\bar{a}$, recalculated using a common slope $\bar{b}$ values of 0.660 and 0.616, respectively, represents *V*o_2_ (mg O_2_ h^−1^ g^−1^) and ${V}_{N{H}_{4}\mbox{--}N}$ (μg NH_4_–N h^−1^ g^−1^) for an individual of 1 g DW. Superscripts indicate significant differences between intercepts (Bonferroni *post hoc* test, *P* < 0.05).

**Figure 4:**
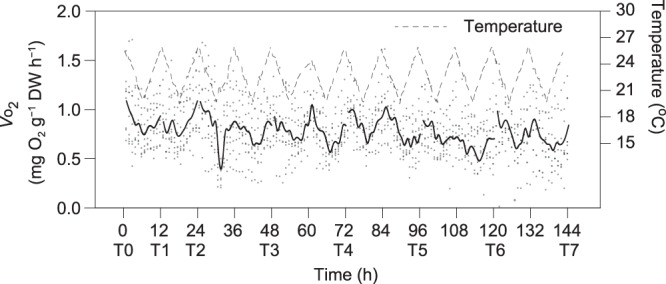
Periodic variation in oxygen consumption rates of *Haliotis discus hannai* measured simultaneously with semidiurnal fluctuations of ambient water temperatures (dashed line) over the 6 d. Points are recalculated regression intercepts from Table 1. The solid line represents the hourly mean values of respiration rates.

For the fluctuating daily temperature (semidiurnal variation) treatments, the rates of oxygen consumption displayed a pronounced semidiurnal pattern in accordance with temperature fluctuation (Fig. 4). ANCOVA revealed no significant differences among the slopes or intercepts of any of the daily *V*o_2_–DW regressions over the 6-day experimental protocol (*F*_6,49_ = 1.559, *P* = 0.179; *F*_6,55_ = 0.904, *P* = 0.499, respectively; Table 1). After recalculation using a common slope of 0.589 (±0.038), no marked changes were found over 6 d in the daily *V*o_2_ of an abalone of 1 g DW (Fig. 3d). These daily *V*o_2_ values were comparable with those in the corresponding stable temperature (e.g. 23 °C) treatment.

A pronounced increase in metabolic rate with increasing test temperature was observed, as indicated by the Q_10_ (8–28 °C) over the entire range of temperatures (Table 2). The Q_10_ displayed reduced thermal sensitivity at the higher temperatures compared with lower temperatures under seasonally acclimated conditions. The Q_10_ (20–26 °C) under the fluctuating daily temperatures was slightly higher than that in the higher temperatures (18–28 °C) of the acclimated conditions.

**Table 2:** Q_10_ values for physiological rates (oxygen consumption and ammonia excretion) of the abalone *Haliotis discus hannai* in stable or fluctuating daily temperatures.

		Metabolic rate	Ammonia excretion rate
Stable daily temperature	Q_10_ (8–28 °C)	10.06	7.03
	Q_10_ (8–18 °C)	3.15	1.48
	Q_10_ (13–23 °C)	1.87	1.36
	Q_10_ (18–28 °C)	1.60	2.37
Fluctuating daily temperature	Q_10_ (20–26 °C)	2.67 ± 0.94	

Note that when Q_10_ = 1, there is no change in physiological rate with changing temperature; when Q_10_ < 1, rates decrease with increasing temperature; and when Q_10_ > 1, rates increase with increasing temperature. At 3 °C, the abalone were quiescent with little metabolic activity and thus this treatment was not considered in the Q_10_ calculation.

### Ammonia excretion by *H. discus hannai*

All regressions of ammonia excretion (${V}_{N{H}_{4}\mbox{--}N}$, μg NH_4_–N h^−1^) against the DW of *H. discus hannai* were significantly positive at both stable and fluctuating temperatures (Table 1). For the stable daily temperature treatments, ANCOVA testing of all these data revealed no significant difference in the slopes for six temperature treatments (*F*_5,42_ = 0.843, *P* = 0.527), with a common slope of 0.616 (±0.081). Significant differences in the intercepts of the regression were found (*F*_5,47_ = 46.193, *P* < 0.001) and a subsequent Bonferroni test demonstrated a significant effect of temperature on excretion rate (*P* < 0.05), revealing an abrupt stepwise increase at the highest test temperature (28 °C) and consistent rates between 8 °C and 23 °C (Fig. 3b).

For the fluctuating daily temperature treatments, there were no significant differences among the slopes or intercepts of any of the daily ${V}_{N{H}_{4}-N}$–DW regressions for 6 d (*F*_6,49_ = 0.439, *P* = 0.849; *F*_6,55_ = 0.957, *P* = 0.463, respectively; Table 1). When recalculated using a common slope of 0.471 (±0.038), the daily excretion rates of an abalone of 1 g DW were fairly consistent for 144 h (6 d) (Fig. 3e), with values comparable to that at the highest stable temperature (28 °C) treatment.

A pronounced thermal sensitivity of the ammonia excretion rate was also observed (Table 2). In contrast to the metabolic rate, the Q_10_ for ammonia excretion increased at the higher temperatures compared with lower temperatures under seasonally acclimated conditions.

### O:N ratio

The ratio of oxygen consumption to ammonia excretion (O:N ratio, by atomic equivalents) increased with increasing test temperature in the stable daily temperature treatments (coefficient of variation [CV] = 68.0). The ratio reached high values of >130 at 13–23 °C but decreased sharply at 28 °C (Fig. 3c). The O:N ratios in the fluctuating daily temperature treatments remained much more consistent (mean of 96, CV = 13.6) during the experimental period of 6 d (Fig. 3f).

### Proteomic responses

**Figure 5:**
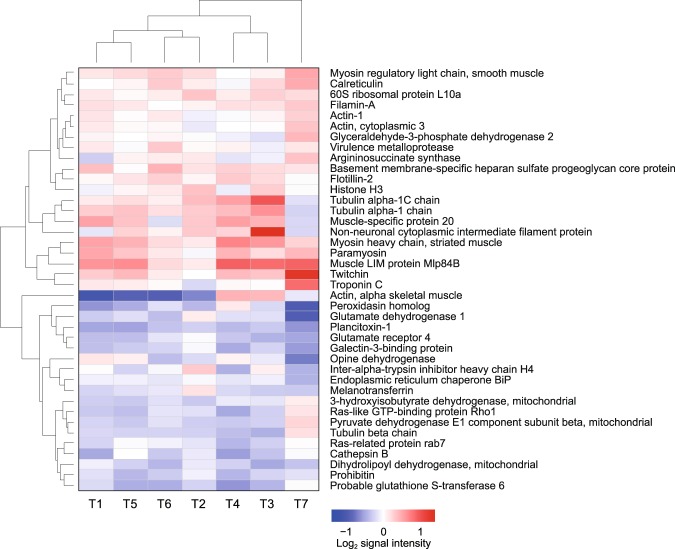
Hierarchical clustering analysis of the differentially expressed proteins in *H. discus hannai* foot muscle exposed to heat stress for different times. Each row indicates the functional annotation of the protein and the columns represent the heat-exposure time points. The up- and downregulated proteins are indicated in red and blue, respectively. A deeper color denotes a larger log_2_-transformed change compared with the control time point (T0).

The iTRAQ-based quantitative analysis of the *H. discus hannai* proteome with triplicates yielded a total of 9,368 spectra; 317 proteins were quantified on the basis of 913 unique peptides. Among these proteins, 217 were identified in all three replicates and 160 proteins were annotated using a BLAST sequence-similarity search (Supplementary Table S1). Among 40 proteins with significantly changed expression compared to the acclimation temperature (26 °C) treatment, 20 were upregulated and 20 were downregulated (Supplementary Table S2). Hierarchical cluster analysis of the changes in protein expression at different time points revealed that although the largest quantitative change occurred at T7, the protein profiles were almost consistent over the rest of the time points (Fig. 5).

**Figure 6:**
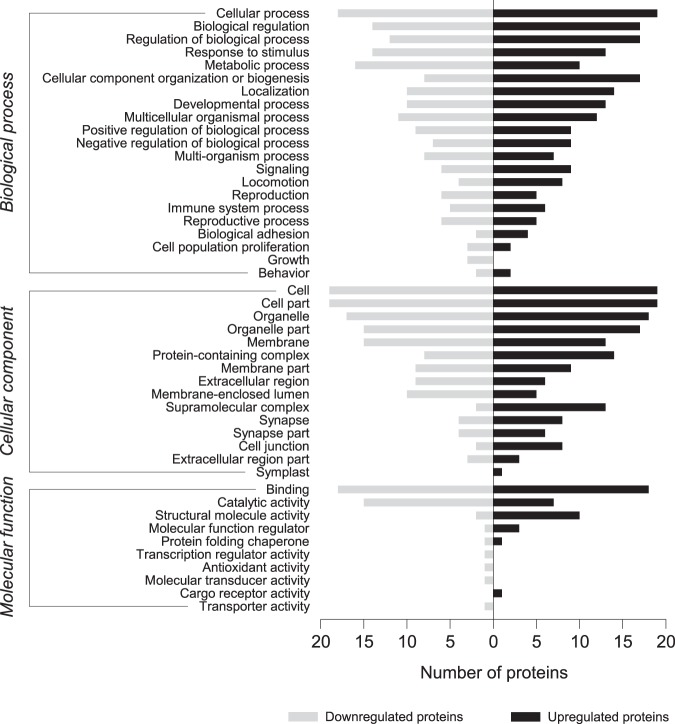
GO analysis of differentially expressed proteins. Each annotated GO term is categorized into biological process, cellular compartment and molecular function. Left-handed bars represent the number of downregulated proteins associated with the corresponding GO term, whereas the right-handed bars indicate the number of upregulated proteins.

The proteins with significantly altered expression were classified based on GO terms. This indicated enrichment of 20, 15 and 9 categories in the biological process, cellular component and molecular function domains, respectively (Fig. 6). The ratio of the numbers of up- and downregulated proteins for each GO term was calculated and a cutoff ratio of 1.5 was applied to enrich up- and downregulated GO terms. For the biological process domain, cellular component organization or biogenesis (GO:0071840), locomotion (GO:0040011) and biological adhesion (GO:0022610) were enriched in the upregulated proteins, whereas metabolic process (GO:0008152) was enriched in the downregulated proteins. With regard to the cellular component domain, upregulated proteins were involved in protein-containing complexes (GO:0032991), supramolecular complexes (GO:0099080), synapses (GO:0045202) and cell junctions (GO:0030054), while downregulated proteins were related to the membrane-enclosed lumen (GO:0031974). For the molecular function domain, structural molecule activity (GO:0005198) and molecular function regulator (GO:0098772) were enriched in the upregulated proteins, whereas catalytic activity (GO:0003824) was enriched in the downregulated proteins.

### Metabolic pathway analysis

A Kyoto Encyclopedia of Genes and Genomes (KEGG) metabolic pathway analysis identified 15 metabolic pathways that were significantly associated (adjusted *P* < 0.05) with up- and downregulated proteins (Supplementary Table S3), of which 14 were related to downregulated proteins and one with upregulated proteins. Among the 14 downregulated pathways, three were related to glucose catabolism and four to amino acid catabolism. Overall, four downregulated proteins were linked to these seven pathways. To visualize the participation of the four downregulated proteins in two different catabolic pathways, two partial pathway maps were generated based on three KEGG pathways of *Lottia gigantea* (owl limpet): pyruvate metabolism (ID:00620), valine, leucine and isoleucine degradation (ID:00280), and alanine, aspartate and glutamate metabolism (ID:00250) (Supplementary Fig. S1). Pyruvate dehydrogenase (PDH) and dihydrolipoyl dehydrogenase (DLD) are associated with the pyruvate catabolism pathway, while 3-hydroxyisobutyrate dehydrogenase (HIBADH) and glutamate dehydrogenase (GDH) are involved in the amino acid catabolism pathway. Among the significantly changed proteins, the pathway analysis found that only GDH was directly correlated with ammonia excretion via catalysis of oxidative deamination of glutamic acid.

## Discussion

The present study demonstrated that large daily temperature fluctuations adjacent to a tidal-mixing front area are episodic events that occur in summer months. We examined the acute physiological responses of abalone to exposure to semidiurnal cyclic fluctuations over a range of environmental temperatures (20–26 °C) rather than using the rate–temperature curves at different exposure temperatures following acclimation to those temperatures^8,34^ or to fluctuating temperatures^10,11^. Abalone experiencing thermal oscillations were unable to reduce the thermal sensitivity of their metabolism compared with those acclimated to the high temperatures of summer–autumn, but their metabolic rates fluctuated in parallel with the temperature fluctuations. In contrast, their ammonia excretion rates were similar between fluctuating and stable high temperatures. Thus, the energy demands required as a consequence of the abalone’s acute response to short-term temperature variations appear to be sustained by an allocation of resources through protein catabolism. These results likely support our initial hypothesis, and suggest that short-term temperature fluctuations will increase the metabolic demands observed at high temperatures in summer. However, contrary to our expectation, different patterns of protein expression in abalone under fluctuating and acclimated (26 °C) temperatures demonstrated a cellular response that reduced mobilization of metabolic reserves under thermally variable conditions, which probably explains why no mortality was observed at the cage site exposed to the largest daily fluctuations in temperature.

In response to an increase in seasonal temperatures, abalone showed no evidence of an acclimatory adjustment of their metabolic rate, which increased sharply during the warm conditions of summer^2,34^. This result is inconsistent with those obtained for other gastropods (e.g. *Crepidula fornicate*^8^ and *Haliotis corrugata*^35^), whose metabolic rate is independent of temperature. Furthermore, the acute response of abalone to thermal fluctuations led to a clear circatidal rhythm in their metabolism coincident with the thermal oscillations (Fig. 4). Indeed, tidal-based endogenous rhythms, similar to the sleeping-and-waking-based circadian rhythms in the metabolism of highly evolved organisms^36^, have been observed in marine ectotherms^37^. However, there is little evidence in marine ectotherms for an endogenous circatidal rhythmicity based on short-term thermal oscillations. Our results demonstrate that the physiological traits of abalone show a strong temperature dependence in response to both seasonal and short-term fluctuations in environmental temperatures.

Metabolism of ectotherms is a major energy-demanding process that provides energy for maintenance, growth, development and performance^2,11^. In this study, we showed that the metabolic costs of abalone are highly temperature dependent. In the absence of mechanisms to reduce the thermal sensitivity of their metabolism^11^ or to compensate for the increased cost of metabolism^2^ during the summer, organisms will experience a metabolic deficit that may suppress maintenance and further growth. It is generally accepted that gastropods become quiescent as environmental temperatures decrease (below 7–8 °C in the case of *H. discus hannai*)^38,39^, allowing them to reduce their metabolic rate. Indeed, higher Q_10_ values indicate that the metabolic rates of abalone are more rapidly reduced at lower temperatures than would be predicted from their response to higher temperatures. The metabolism of abalone acclimated to high temperatures (18–28 °C) displayed reduced thermal sensitivity compared with those at low temperatures and across a range of temperatures; this may be a mechanism by which abalone buffer their high metabolic costs during the peak temperatures of summer. In contrast, although abalone exposed to fluctuating temperatures showed little evidence of a reduction in the thermal sensitivity of their metabolism at stable high temperature, the thermal sensitivity of their metabolism was still much lower in fluctuating temperatures than that across the entire range of temperatures experienced. This may result from a daily metabolic rate that is broadly comparable with that predicted by the mean temperature during thermal oscillations.

During both acclimation to different temperatures and exposure to fluctuating thermal conditions, temperature affected the ammonia excretion rate of abalone. Without a compensatory adjustment (e.g. an increase in food consumption), the increased energy demands of abalone exposed to high temperatures and thermal oscillations may deplete their tissue energy in summer. Our finding that abalone experience food shortages under cage-cultivation conditions was unexpected. Their food consumption is temperature dependent, increasing with temperature to 20 °C and decreasing above this temperature^38,40^. Given that our physiological measurements were conducted in starved abalone, our results for metabolic and excretory rates would represent the basal requirements for maintenance under conditions of restricted food consumption at high or fluctuating temperatures in summer. In both cases, elevated ammonia excretion will give rise to a depletion of tissue energy reserves because of increased maintenance requirements and the resultant mismatch between metabolic demand and energy intake, resulting in disturbed maintenance and a reduction in growth.

The O:N ratio represents the relative proportion of resources (proteins *vs*. carbohydrates and lipids) catabolized for maintenance metabolism by marine ectotherms^2,41^. The O:N ratio displayed a sudden decrease at the highest temperature studied (28 °C). Given that lowered O:N ratios indicate a relative enhancement of protein catabolism compared with overall oxidative energy metabolism^41^, stable high-temperature stress caused a metabolic shift from carbohydrate and lipid catabolism to protein catabolism. The daily mean O:N ratios of abalone exposed to fluctuating temperature regimes were comparable with that at stable high temperature (28 °C). However, the acute change in metabolic rates in accordance with temperature fluctuations suggest that it is reasonable to infer that the greater thermal sensitivity of ammonia excretion at high temperatures may make O:N ratios highly variable during thermal oscillations. Although the mean values of *V*o_2_, ${V}_{N{H}_{4}\mbox{--}N}$ and O:N ratios were comparable in fluctuating temperatures and at stable high temperature, these increased costs may have been associated with the high temperatures experienced during thermal fluctuations. Evidence for such responses of physiological traits to short-term thermal fluctuations comes from the different patterns of synthesis and utilization of reserves as evidenced by different protein profiles in abalone exposed to stable and fluctuating temperatures, as discussed below.

One of the most interesting results of this study was that there was a notable discrepancy between protein expression in abalone exposed to fluctuating temperatures and those exposed to high acclimation temperature (26 °C). The hierarchical clustering analysis of the abalone proteome under exposure to fluctuating temperatures revealed limited changes during the experimental period and differential expression compared with the control (Fig. 5). The GO analysis of the molecular function category revealed that a large proportion of downregulated proteins (75%) was associated with the catalytic activity term (GO:0003824) and half (50%) the upregulated proteins were associated with the structural molecular activity term (GO:0005198) (Fig. 6). These results imply that the majority of downregulated proteins are metabolic enzymes, whereas the majority of upregulated proteins are structural. It is thus not surprising that the largest number of significantly changed KEGG metabolic pathways were correlated with downregulated proteins (Supplementary Table S3). In this context, of four downregulated proteins (PDH, DLD, HIBADH and GDH), only GDH, which produces energy through deamination of glutamate to α-ketoglutaric acid, had a direct effect on the rate of ammonia excretion (Supplementary Fig. S1). Given that the deamination cycle of GDH is known to be a major pathway of ammonia excretion in marine invertebrates^42^, the mean O:N ratio observed under the fluctuating temperature stress may reflect the result of a downregulation of GDH expression below that seen at the high control temperature.

The analysis of the time course of changes in protein expression under fluctuating temperatures demonstrated a significant decrease of GDH at the time point T7 (Supplementary Fig. S2a). To investigate the correlation between the time course of expression of GDH and muscle constituents, a pairwise Spearman’s correlation analysis was performed between GDH and eight proteins annotated with the GO term of structural molecule activity. Among these eight muscle-related proteins, four proteins that are major components of muscle myofibril^43^ showed a strong negative correlation with GDH expression (Spearman’s ρ < −0.7) (Supplementary Fig. S2b). With respect to dynamic nitrogen homeostasis, muscle myofibril can act as an ‘amino acid deposit’, because it is synthesized under high concentrations of amino acids and degraded under low amino-acid concentrations^44^. The lysosome pathway is considered a major route of protein degradation in muscle tissue, and given that it is significantly downregulated under fluctuating temperature stress (adjusted *P* < 0.05) (Supplementary Table S3), suppressed lysosomal proteolytic activity is likely to be responsible for the increased expression of muscle myofibril proteins^45^.

The significant negative correlations between GDH and muscle myofibril proteins can be explained by suppression of lysosomal proteolysis because of an increased free amino acid concentration as a result of downregulation of GDH. Cellular free amino acids in muscle tissue are considered to be major regulators of lysosomal proteolysis^46^ via the mammalian target of the rapamycin (mTOR) protein complex-mediated signaling pathway^47,48^. Glutamine and arginine, which are negatively regulated by glutamate dehydrogenation of GDH^49,50^, are known to positively regulate mTOR phosphorylation, leading to a downregulation of lysosome-mediated proteolysis^51,52^. Thus, the downregulation of GDH in abalone exposed to fluctuating temperatures may result in high concentrations of free glutamine and arginine in their muscle cells, shifting the net balance of protein metabolism from catabolic to anabolic. Additional quantitative analyses would offer more solid evidence of the time-course correlation between GDH downregulation and muscle myofibril proteins. For instance, quantitation of free amino acids including glutamine and arginine could demonstrate the correlation between GDH and amino acid catabolism, and quantitative phosphoproteome analysis of the mTOR-related pathway could provide evidence of altered lysosomal proteolysis activity.

The metabolic pathway analysis provided a plausible explanation for the correlation between physiological properties and differential protein expression. The downregulation of metabolic enzyme activity (PDH, DLD, HIBADH and GDH) in abalone exposed to fluctuating temperatures did not correspond with the high metabolic rate that would be predicted by the mean temperature during thermal oscillations or at high control temperature. This indicates high maintenance costs and suggests that exposure to low temperatures during thermal oscillation alters metabolic enzyme activity, inducing a reduction in metabolism and protein catabolism compared with that at the high control temperature. The consistency between the responses of physiological processes and metabolic enzyme activity to fluctuating temperatures highlights a close interaction between physiological responses and cellular-level molecular processes. Together with downregulation of metabolic enzymes, upregulation of structure-related proteins may provide a greater metabolic scope that would help meet the metabolic demands for growth under fluctuating temperatures^10^. Therefore, when abalone are fed in cultivation cages, the energetic consequences of exposure to low temperatures during thermal oscillations may have a synergistic effect on growth, with the most active feeding capacity being at around 20 °C^38,40^. Accelerated growth under daily fluctuating temperature conditions has been clearly demonstrated for other marine ectotherms including fish and invertebrates^15,20^.

To conclude, this study reveals a potential mechanism by which exposure to fluctuating temperatures allows abalone to deal with the challenges associated with summer temperature extremes. The abalone exhibited the capability to regulate protein expression and thereby adjust their physiological traits to fluctuating temperatures in a way that allows them to reduce the elevated energetic costs of summer peak temperatures. As previously reported for temperature stress^27^, exposure to high temperatures of more than 26 °C for several days severely affected the survival, falling rate, histological structure of the foot, and antioxidant and stress-response systems of *H. discus hannai*, whereas no biological and molecular effects were detectable in abalone exposed to temperatures lower than 22 °C. Such biological responses to high-temperature stress were dependent upon the exposure time. In this context, fluctuating thermal regimes may mitigate the potential biological risk of summer peak temperatures by reducing exposure time and increasing the upper thermal limits^10^. Overall, our results suggest that acute regulation of physiological traits and protein expression by semidiurnal thermal fluctuations may buffer abalone from the lethal consequences of extreme temperatures in summer and actually allow their long-standing cultivation.

## Methods

### Sample collection, acclimation and experimental design

Additional details of the abalone aquaculture at the sampling site are given in the supplementary material.

Specimens of the Pacific abalone *Haliotis discus hannai* for the experiments were collected in a cultivation facility off Wando Island, on the southwestern coast of Korea (34°18′N and 126°63′E). Because of the known mortality of the cultivated abalone and to establish the experimental conditions, seawater temperatures were measured in 2017 at three culture cages with different degrees of daily temperature variation throughout the year. The three sites included a moderate-mortality site (MM), a high-mortality site (HM) and a no-mortality site (NM). Temperature data were recorded every half hour using a HOBO Water Temperature Pro v2 Data Logger (Bourne, MA) installed in the submerged culture facility (http://www.nifs.go.kr/risa/). The numbers of individuals that survived in the cultivation cages were counted every month to estimate monthly mortality at the geographical locations of the study area.

Because of the regular seasonal fluctuation of water temperature in this area, 40 specimens on each seasonal sampling occasion were randomly collected in May, August and November 2017, and February 2018. Immediately after collection, all of the individual abalone were put into a container of natural seawater saturated with oxygen by a portable air generator and transferred to 200 L water tanks in the laboratory. The tanks were filled with filtered seawater with a continuous flow system and aeration. 20 animals were gradually acclimated to the experimental temperatures (3, 8, 13, 18, 23 or 28 °C, respectively) covering the seasonal variation observed *in situ* and the temperature change was controlled at a rate of 1 °C per day to minimize the potential effects of acute temperature changes on physiological state (Fig. 1a). Specimens for the daily fluctuating treatment were kept under summer temperature (26 °C) (Fig. 1b). All abalone were kept on a 14 h:10 h (light:dark) photoperiod. During the 7-day acclimation to ensure full thermal adaptation to the fortnightly spring–neap tidal cycle, the abalone were fed fresh sea mustard (*Undaria pinnatifida*) daily and then were starved for 24 h prior to the experiments to reduce the associated physiological responses. The animals were transported to experimental chambers and acclimated again for 6 h under the chamber conditions before the commencement of measurements of physiological activities.

For stable daily temperature (seasonal variation) experiments, 350 ml flow-through closed cylindrical chambers equipped with a magnetic stirrer were used to measure the oxygen consumption and urine excretion rates of the abalone. Nine individual experimental chambers each accommodated a single abalone and one was used as a control without an abalone. Seawater was continuously supplied to individual chambers from an aerated water tank by a peristaltic pump equipped with a 10-channel pump head (BT 100–1 L, Longer Precision Pump Co. Ltd, Baoding, China). Flow rates in individual chambers were adjusted to approximately 20 ml min^−1^ to maintain the oxygen concentration at over 80% saturation^53^. For each experiment, the chambers were completely immersed in a water bath in which water temperature was kept constant at that required for individual experiments (3, 8, 13, 18, 23 or 28 °C; Fig. 1a). Physiological measurements were performed over 24 h. After the experiments, the specimens were carefully dissected using a stainless steel knife. Shells were dried at 60 °C in a drying oven. Soft tissues were lyophilized and then weighed.

For fluctuating daily temperature treatments, two series of experiments were performed. Nine individual experimental chambers and one control chamber, as described above, were placed in a water bath for physiological measurements and approximately 50 abalone were placed in another water tank for measurements of molecular responses. The experimental temperature was set to range between 20 and 26 °C (Fig. 1b), based on previous observations of daily temperature variability in the abalone culture cages^25^. A comparison of temperature data reading at half-hour intervals at two locations adjacent to our sampling sites from 2005 to 2009 showed that mean values and fluctuating ranges of daily temperatures fluctuation were highest in August. The full range of daily temperatures was much larger (up to 8.5 °C) in deep offshore area, where tidal front is formed, than that (<1 °C) in the shallow coastal area. The daily temperature ranges were closely associated with fortnight tidal cycle and horizontal temperature gradient. To reproduce the thermal variability during semidiurnal tidal cycles, the water temperature in the water baths was changed periodically at intervals of 6 h, increasing or decreasing by 0.3–0.4 °C every 20 min. Because of the observed period of high daily temperature fluctuations in summer^25^, physiological measurements for the fluctuating temperature treatments were performed over 144 h. The specimens were carefully dissected for biometric measurements as described above. For proteomic analysis, three specimens were randomly sampled from the water tank in which 50 abalone were situated at each time point of 0, 12, 24, 48, 72, 96, 120 and 144 h (T0–T7, respectively) after the experiment started.

### Physiological measurements

The metabolic rate of the abalone was determined by measuring oxygen consumption inside the control and experimental chambers. Oxygen concentrations inside the measurement chambers were recorded every second using oxymetric probes (Oxy-10 micro, PreSens-Precision Sensing GmbH, Regensburg, Germany). The measurements were performed for 24 h (for constant temperature treatments) or 144 h (for fluctuating temperature treatments). The oxygen consumption of the specimens was then calculated from the differences in oxygen concentration between the control and the experimental chambers. Details of the procedures used to determine oxygen consumption by continuous monitoring have been described elsewhere^54^.

The rate of ammonia excretion was determined from the increase in ammonia concentrations between the outflows of the control and experimental chambers. Water samples from the outflows of the chambers were collected 4–5 times during the 24-hour experiments. Ammonia concentration was analyzed using the oxidation method with hypochlorite in an alkaline medium^55^.

### Statistics of physiological data

Differences in the daily fluctuations of water temperatures for abalone rearing cages were tested by an analysis of variance (ANOVA) after checking normality (Shapiro–Wilk test) and homogeneity of variance (Levene’s test).

The physiological measures (i.e. rates of oxygen consumption and ammonia excretion) and dry weight (DW) of the abalone for individual experimental conditions were fitted to the allometric equation: $Y=a{W}^{b}$, where *Y* is the physiological rate, *W* is DW, and *a* and *b* are the fitted constants. The fitted constants were determined by the intercepts (*a*) and slopes (*b*) of least-squares regression between physiological rates and DW values of the specimens based on the logarithmic transformation (base 10) of those variances. The significance of the differences in slopes and intercepts of regression equations was tested by analysis of covariance (ANCOVA). When no significant differences were detected among these estimated slopes (*P* > 0.05), the intercepts were recalculated using a common slope ($\bar{b}$). The intercepts of the various sets of regression equations were compared by a Bonferroni *post hoc* multiple-comparison test. The commercially available SPSS software package (v. 22.0, IBM Corp., Armonk, NY) was used to perform the statistical analyses.

The thermal sensitivity of physiological rates for metabolism and ammonia excretion were calculated as ${Q}_{10}={({R}_{2}/{R}_{1})}^{(10/({T}_{2}-{T}_{1}))}$, where *R* represents the rates at temperature (T) 1 and 2. Thermal sensitivities were calculated for the entire range of test temperatures, the lower (8–18 °C) to upper (18–28 °C) temperature ranges and for the fluctuating daily temperature range.

### Proteomic analysis

Details of the procedures for proteomic analysis are presented in the supplementary material. Immediately after collection of triplicate specimens for determination of cellular molecular responses to semidiurnal temperature fluctuation, the foot tissues of specimens were rapidly excised and frozen in liquid nitrogen (−80 °C). After purification and digestion of cellular proteins, equal amounts of peptides (50 μg) from each replicate were treated for iTRAQ labeling. Peptides derived from samples at each time point, T0–T7, were labeled with iTRAQ tags 113, 114, 115, 116, 117, 118, 119 and 121, respectively. The iTRAQ labeling peptides were fractionated by an in-house nano-LC system interfaced with a Q-Exactive Hybrid Quadrupole-Orbitrap Mass Spectrometer equipped with a preconditioned capillary column (0.075 × 150 mm, C18, 3 μm particle size, 200 Å pore size, Phenomenex, Torrance, CA).

Proteome Discoverer version 2.1 (Thermo Scientific, Waltham, MA) was used to search the peptide MS/MS data against the ORF-based protein database of *H. discus hannai*^56^. A peptide sequence was assigned to the tryptic peptide sequence using the SEQUEST search engine. For the entire peptide ID list, the false discovery rate (FDR) was calculated by applying the PeptideProphet software using a reverse decoy database and a discriminant score cutoff was set to an FDR of less than 1% to identify peptides. The identified proteins were relatively quantified based on the iTRAQ reporter ion intensity corresponding to each sampling time point by comparing intensity at a time point Tn (n = 1–7) (I(Tn)) to that of the control (I(T0)). The functional annotation of proteins was performed by BLAST sequence similarity searches against the whole-organism UniProt/Swiss-Prot database (E-value < 1 × 10^−20^). The protein fold change at the nth time point (Tn/T0) was calculated for the time points from T1 to T7. To identify significant changes, the following criteria were applied: Student’s *t*-test over biological triplicates (*P* < 0.05) and the absolute cutoff of an average fold change >1.20 or <0.83 for up- or downregulated proteins, respectively. Log_2_-transformed iTRAQ ratios were used to evaluate differential protein expression^57^ and were visualized by a hierarchical clustering heat-map matrix. The biological processes and molecular functions of the regulated proteins were functionally annotated in the context of their gene ontology (GO) using Blast2GO (v. 5.2.5, BioBam, Valencia, Spain)^58^. Furthermore, Kyoto Encyclopedia of Genes and Genomes (KEGG) pathway enrichment analysis for differentially expressed proteins was performed in KOBAS 3.0^59^ to identify significantly enriched pathways.

## Supplementary information

Supplementary Information

Supplementary Table S1

Supplementary Table S2

Supplementary Table S3
