# Supplementary material for: Physiological responses of the abalone Haliotis discus hannai to daily and seasonal temperature variations
Source: Sci Rep. 2019 May 29;9:8019. doi: 10.1038/s41598-019-44526-3 (PMC6541628; doi:10.1038/s41598-019-44526-3)
Supplement: Supplementary file 1 — Supplementary Information [file 41598_2019_44526_MOESM1_ESM.docx]

**Supplementary Information**

Physiological responses of the abalone *Hailotis discus hannai* to daily and seasonal temperature variations

Hee Yoon Kang, Young-Jae Lee, Woo-Young Song, Tae-Ik Kim, Won-Chan Lee, Tae Young Kim & Chang-Keun Kang

**Methods: Description of sampling site and proteomic analysis**

**Description of sampling site.** The shallow coastal sea around the archipelago off southwestern Korea represents one of the most intensive areas of abalone cultivation in Korea, the annual production of which accounts for about 80% of overall domestic production (i.e. about 8,000 tonnes in 15.7 km^2^ in this study). The size of cultivation cages is 2.4 m × 2.4 m × 2.5 m (height × width × depth) and initial stocking density was in the range of 1000–2000 individuals depending on abalone size. Sea mustard (*Undaria pinnatifida*) or sea tangle (*Laminaria japonica*) are used as feed for the cultivated abalone. More details of the abalone aquaculture in this area have been reported elsewhere^1^. The water temperature of the coastal sea around the archipelago ranges from 5.5 °C in February to 28.7 °C in August^2^. The tide in this area is semidiurnal with a tidal amplitude of 4.1 m for spring tides and 1.1 m for neap tides (http://www.khoa.go.kr/koofs/kor/). Strong tidal currents and bottom friction in this shallow area induce vertical mixing of the water column and form a tidal front at a depth of 20–40 m, especially in summer^3,4^. The resultant colder surface water flows toward the coast line, leading to large daily temperature fluctuations (up to 8 °C) in the abalone cultivation cages^2^.

**Protein preparation and iTRAQ labeling.** Immediately after collection of specimens for determination of cellular molecular responses to semidiurnal temperature fluctuation, their foot tissues were rapidly excised and rinsed with Milli-Q water to remove salts. Washed tissues were then frozen in liquid nitrogen and stored at −80 °C until analysis. Fifty milligrams of each foot tissue were resuspended in 1 ml lysis buffer (8 M urea, 50 mM Tris at pH 8, 0.1% SDS, 1× solution of MS-SAFE protease inhibitor, Merck, Darmstadt, Germany) and homogenized on ice for 30 s using an Omni TH homogenizer (Omni International, Kennesaw, GA). The homogenized sample was incubated on ice for 20 min and centrifuged at 13,500 rpm for 20 min at 4 °C. Cellular proteins were precipitated by adding 800 μl of cold acetone (−20 °C) to 200 μl of the tissue lysate supernatant. The purified protein sample was dissolved in 600 μl of digestion buffer (50 mM Tris buffer with 2 M urea at pH 8), and 10 μl of the protein solution was mixed with 200 μl of bicinchoninic acid (BCA) reagent (Thermo Scientific, Waltham, MA). For calibration, a bovine serum albumin standard solution (Merck) at seven different concentrations ranging from 4 to 0.01 μg μl^−1^ was mixed with BCA reagent in the same manner. The mixtures were incubated at 37 °C for 30 min and quantified using a Nanodrop 2000 UV absorption spectrophotometer (Thermo Scientific, Waltham, MA) at 562 nm. After determining the protein concentration, each solution was diluted to 1 μg μl^−1^ using the digestion buffer.

Fifty microliters of protein solution (1 μg μl^−1^) were reduced by adding 5.6 μl of 50 mM dithiothreitol (Merck) for 60 min at 37 °C, followed by alkylation with 4.3 μl of 280 mM iodoacetamide (Merck) for 60 min at room temperature in the dark. Subsequently, the samples were digested with 1 μg of sequencing-grade trypsin (Promega, Madison, WI) at 37 °C for 16 h. The digested samples were desalted using Sep-Pak C18 cartridges (Waters, Milford) and dried in a SpeedVac (Centrivap vacuum concentrator, Labconco, Kansas City, MO). Fifty micrograms of each peptide sample were reconstituted in 30 μl of 0.5 M triethylammonium bicarbonate (Merck) and 50 μl of isopropyl alcohol (Fisher Scientific, Waltham, MA). Each sample was labeled with iTRAQ reagents for 2 h at 25 °C following the manufacturer’s instructions (Applied Biosystems, Framingham, MA). Peptides derived from each sampling time point, T0–T7, were labeled with the iTRAQ tags 113, 114, 115, 116, 117, 118, 119 and 121, respectively. The labeled peptides were pooled and purified using Oasis HLB cartridges (Waters, Milford) to remove the unreacted labeling reagents.

**LC–MS/MS analysis.** The iTRAQ-labeled peptide samples were reconstituted with 200 μl of 0.1% formic acid (Merck). Ten microliters of each sample were injected into the in-house nano-LC system interfaced with a Q-Exactive Hybrid Quadrupole-Orbitrap Mass Spectrometer (Thermo Scientific, Bremen, Germany). The peptides were separated at a flow rate of 250 nl min^−1^ on a preconditioned capillary column (0.075 × 150 mm, C18, 3 μm particle size, 200 Å pore size, Phenomenex, Torrance, CA). The mobile phase was composed of solvent A (0.1% formic acid in water) and solvent B (0.1% formic acid in acetonitrile, Thermo Scientific, Bremen, Germany). A 120 min gradient was applied as follows: 100 min of 5–55% mobile phase B, 10 min of 55–80% B and 10 min of 80% B. The electrospray potential was set to 4.0 kV. The mass spectrometer was set to operate in the positive ion mode with a full MS scan (resolution 70,000 at m/z 400; 350–1600 m/z range) in a profile mode. Subsequently, the MS/MS spectra were recorded at a resolution of 17,500 by fragmenting the 10 most intense ions using the higher energy collision dissociation (HCD) at a normalized collision energy of 30 eV.

**Proteome data analyses.** Proteome Discoverer version 2.1 (Thermo Scientiﬁc) was used to compare the peptide MS/MS data against the ORF-based protein database of *H. discus hannai*^5^. Each MS/MS spectrum was assigned to a tryptic peptide sequence using the SEQUEST search engine. The allowed number of missed tryptic cleavages was one. iTRAQ 8-plex modification (peptide N-term, K and Y), protein N-terminal acetylation, oxidation (M) and phosphorylation (S, T and Y) were set as dynamic modifications, with carbamidomethylation (C) as a static modification. For the entire peptide ID list, the false discovery rate (FDR) was calculated by applying the PeptideProphet software using a reverse decoy database. As a result, the discriminant score cutoff was set to an FDR < 1%. Proteins having at least one unique peptide with a score above the discriminant cutoff were considered to be identified. The proteins identified in all three replicates were subjected to the following quantitative analysis.

The identified proteins were relatively quantified based on the iTRAQ reporter ion intensity corresponding to each sampling time point. First, the reporter ion groups in all peptide–spectrum matches (PSMs) were summed to yield a peptide reporter ion group. From comparison of peptide reporter ion intensity at a time point Tn (n = 1–7) (I(Tn)) to that of the control (I(T0)), the fold change in the abundance of each unique peptide (I(Tn)/I(T0)) was calculated. Lastly, the fold change in the protein expression levels was obtained by weighted averaging of the fold changes of all unique peptides belonging to a specific protein with their reporter ion intensities^6^. The functional annotation of proteins was performed by BLAST sequence similarity searches against the whole-organism UniProt/Swiss-Prot database (E-value < 10^−20^). The protein fold change at the nth time point (Tn/T0) was calculated for each time point from T1 to T7. To identify significant fold changes, the following criteria were applied: Student’s *t*-test over biological triplicates (*P* < 0.05) and the absolute cutoff of an average fold change > 1.20 or < 0.83 for up- or downregulated proteins, respectively. Proteins exhibiting a significant fold change for at least one time point were considered to be differentially expressed (no protein showed bidirectional changes in abundance over different time points).

To examine the time-course changes in protein expression of significantly changed proteins, a complete-linkage hierarchical cluster analysis was conducted on the log_2_-transformed fold-change values using Genesis 1.8.1^7^, and the results were visualized by a heat-map matrix. A gene ontology (GO) analysis was performed to identify the biological process, cellular component and molecular function categories of the differentially expressed proteins. The list of up- and downregulated proteins was loaded into Blast2GO software (v. 5.2.5, BioBam, Valencia, Spain)^8^, where GO mapping and annotations were performed (E‐value hit filter ≤ 10^−5^; annotation cutoff = 55; GO level = 2, GO weight = 5; HSP‐hit coverage cutoff = 0)^9^. To identify the metabolic pathways related to differentially expressed proteins, a pathway analysis was performed against the KEGG database of *Lottia gigantean* (owl limpet), which is taxonomically the closest gastropod to the abalone, using a KOBAS 3.0 server^10^. The statistical significance of each pathway was assigned by a hypergeometric test with FDR correction developed by Benjamini and Hochberg^11^.

**References**

1. Son, M. H., Park, M. W., Kim, K. W., Kim, K. D. & Kim, S. K. Status of the abalone (*Haliotis discus hannai*) aquaculture for optimal rearing technique in marine net cage. *J. Fish. Mar. Sci. Edu.* **22**, 362–373 (2010).
2. Yang, J. Y., Lee, J. S., Han, I. S., Choi, Y. K. & Suh Y. S. Seawater temperature variation at aquafarms off Wando in the southwest coast of Korea. *J. Korean Soc. Mar. Environ. Safe.* **18**, 514–519 (2012).
3. Cho, Y. K., Choi, B. H. & Chung, H. W. Variation of tidal front in the southwestern sea of Korea. *J. Korean Soc. Coast. Ocean Eng.* **7**, 170–175 (1995).
4. Jeong, H. D., Kwon, C. H., Kim, S. W. & Cho K. D. Fluctuation of Tidal Front and Expansion of Cold Water Region in the Southwestern Sea of Korea. *J. Korean Soc. Mar. Environ. Safe.* **15**, 289–296 (2009).
5. Nam, B.-H. *et al*. Genome sequence of Pacific abalone (*Haliotis discus hannai*): the first draft genome in family Haliotidae. *GigaScience* **6**, gix014 (2017).
6. Li, Z. *et al*. Systematic comparison of label-free, metabolic labeling, and isobaric chemical labeling for quantitative proteomics on LTQ Orbitrap Velos. *J. Proteome Res.* **11**, 1582–1590 (2012).
7. Sturn, A., Quackenbush, J. & Trajanoski, Z. Genesis: cluster analysis of microarray data. *Bioinformatics* **18**, 207–208 (2002).
8. Conesa, A. *et al.* Blast2GO: a universal tool for annotation, visualization and analysis in functional genomics research, *Bioinformatics* **21**, 3674–3676 (2005).
9. Garczynski, S. F. *et al*. Application of *Cydia pomonella* expressed sequence tags: Identification and expression of three general odorant binding proteins in codling moth. *Insect Sci.* **20**, 559–574 (2013).
10. Xie, C. *et al*. KOBAS 2.0: a web server for annotation and identification of enriched pathways and diseases. *Nucleic Acids Res.* **39**, W316–W322 (2011).
11. Benjamini, Y. & Hochberg, Y. Controlling the false discovery rate: a practical and powerful approach to multiple testing. [*J. R. Stat. Soc. B.*](https://www.ncbi.nlm.nih.gov/nlmcatalog?term=%22J+R+Stat+Soc+Series+B+Stat+Methodol%22%5bta%5d) **57**, 289–300 (1995).

**Supplementary Table S1.** List of total proteins identified and quantified by LC-MS/MS analyses using an 8-plex iTRAQ approach.

**Supplementary Table S2.** List of differentially expressed proteins identified and quantified by LC-MS/MS analyses using an 8-plex iTRAQ approach.

**Supplementary Table S3.** The pathways associated with significantly changed proteins with Benjamini–Hochberg corrected *P* values generated by KOBAS. Databases: KEGG PATHWAY; statistical test method: hypergeometric test/Fisher’s exact test; FDR correction method: Benjamini and Hochberg. ^a^*P* values are corrected for multiple comparisons using the Benjamini–Hochberg method (*P* < 0.05).

**Supplementary Figure S1.** Partial metabolic pathway map of (a) pyruvate decarboxylation and (b) amino acid catabolism reconstructed from KEGG pathway of *L. gigantean*. Green boxes represent the proteins downregulated under the influence of semidiurnal temperature fluctuations, whereas white boxes denote unidentified proteins. The initial substrates of partial pathways are marked in blue. Oxygen and ammonium ions are marked in red with arrows representing decreased respiration and ammonia excretion rates, respectively, under the exposure to semidiurnal temperature fluctuation.

**Supplementary Figure S2.** Log_2_-transformed fold changes of (a) glutamate dehydrogenase 1 and (b) four muscle constituent proteins that showed a significant time-dependent negative correlation (Spearman’s ρ < −0.7).
